# Unusual Vestibulo-Ocular Reflex Responses in Patients With Peripheral Vestibular Disorders Detected by the Caloric Step Stimulus Test

**DOI:** 10.3389/fneur.2020.597562

**Published:** 2020-11-30

**Authors:** Motomu Honjo, Keiji Honda, Takeshi Tsutsumi

**Affiliations:** ^1^Department of Otolaryngology, Tsuchiura Kyodo General Hospital, Ibaraki, Japan; ^2^Department of Otolaryngology, Tokyo Medical and Dental University, Tokyo, Japan

**Keywords:** caloric test, peripheral vestibular disorders, vestibulo-ocular reflex, vestibular adaptation, time constant

## Abstract

The caloric step stimulus test consists of the changes in head position from the sitting to supine positions and continuous caloric irrigation. This test can provide a single labyrinth with a stimulus similar to constant head acceleration in rotational testing and, therefore, can evaluate vestibulo-ocular reflex (VOR) dynamics more precisely than can conventional methods. To assess the clinical utility of the test in the assessment of the VOR dynamics of diseases, we performed the test in patients with peripheral vestibular disorders, including sudden idiopathic hearing loss, vestibular neuritis, Meniere disease, vestibular Meniere disease, or chronic unilateral idiopathic vestibulopathy and normal controls. Slow-phase eye velocity (SPV) was measured with videonystagmography. We fitted the time course of SPV across 2 min to a mathematical model containing two exponential components and time constants: the caloric step VOR time constant (*T*_1_) and caloric step VOR adaptation time constant (*T*_2_). All responses of normal controls (*n* = 15 ears) were fit to the model. Several responses of the 101 ears of the patients differed from the time courses predicted by the model. We divided the data of 116 ears into four patterns based on SPV, *T*_1_, and *T*_2_. The thresholds for the classification were determined according to the lower limits of the capability of curve fitting for SPV and the upper limits of normal controls for *T*_1_ and *T*_2_. Seventy-eight ears followed pattern A (normal *T*_1_ and *T*_2_): the SPV trajectory formed a rapid rise with subsequent decay. Nineteen followed pattern B (normal *T*_1_ and prolonged *T*_2_): the SPV trajectory formed a rapid rise without decay. Six followed pattern C (prolonged *T*_1_ and *T*_2_): the SPV trajectory formed a slow rise. Thirteen ears followed pattern D: a low VOR response. There were no significant differences in time constants between the affected and healthy ears in patients with each disease. However, prolonged *T*_1_ and *T*_2_ were significantly more frequent in the affected ears than the healthy ears. In conclusion, the caloric step stimulus test can be potentially useful in detecting unusual VOR responses and thus reflect some pathological changes in the vestibular system.

## Introduction

Used worldwide to assess patients with vestibular dysfunction, the caloric test can evaluate the low-frequency horizontal vestibulo-ocular reflex (VOR) in each ear. In the conventional bithermal caloric test, cold or warm water is irrigated into the external ear canal to create a temperature gradient to the lateral semicircular canal (SCC). The test requires subjects to lay on a bed in a supine position with approximately 30° of neck anteflexion such that the lateral SCC is positioned in the vertical plane. Various theories describing the mechanism of the caloric test have been proposed, including endolymphatic natural convection flow on earth, expansive convection in microgravity, and direct thermal inhibition of ampullary nerve activity (1, 2). Recent studies have suggested that the dominant mechanism of caloric stimulation under gravity is the pressure change due to the buoyancy of the endolymph (3–5).

The conventional caloric test is incapable of accurately evaluating the parameters of the VOR dynamics model, such as the time constant, because the precise onset time of the deflection of the cupula is unmeasurable due to the slow induction of the thermal gradient. The VOR time constant is usually obtained by measuring horizontal slow-phase eye velocity responses to angular rotation during rotational testing. The behavioral VOR time constant is approximately two- to three-fold that of the cupula or vestibular nerve, which is estimated to be 4–6 s. The prolongation is accomplished by “the velocity-storage mechanism” in the central nervous system (6–8). The VOR adaptation to sustained labyrinthine stimulation can also be analyzed, and the time constant can be estimated from constant head acceleration (9–12) or magneto-hydrodynamic stimulation (13).

Formby and Robinson (14) introduced a new procedure for quantitative caloric testing, “caloric step stimulus,” to evaluate the caloric step VOR (VOR_cs_) and VOR_cs_ adaptation time constants by combining rapid head position changes with continuous caloric irrigation. The procedure benefits from not requiring high-cost devices, such as a rotational chair or a 7T-MRI, and its capability of evaluating VOR function independently for a single labyrinth. This protocol consists of two phases: a neutralization and an acceleration phase. In the former, caloric irrigation is performed while the subject sits and leans his or her head slightly forward to position the lateral SCC in the horizontal plane with respect to gravity; in this position, a steady-state thermal gradient is established across the lateral SCC, preventing the cupula from being deflected by the thermal stimulation. The acceleration phase is achieved by rapidly moving the head to the supine position; this shift in position placing the lateral SCC in the vertical plane and consequently induces a pressure change in the cupula that is effected by thermal buoyancy. By changing from the sitting to the supine position or vice versa, we can achieve vestibular stimulation of any duration. Formby and Robinson considered stimulations of caloric step stimulus were theoretically equivalent to those of constant head acceleration in rotational testing, although rotational testing stimulates both labyrinths and caloric test does single labyrinth. In fact, the resulting caloric step VOR responses were similar to rotatory acceleration step responses (9, 10).

Estimating the VOR_cs_ and VOR_cs_ adaptation time constants can be potentially useful in the evaluation and diagnosis of peripheral and central vestibular disorders. Rotational testing revealed that the VOR time constant is reduced in unilateral vestibular disorders (15). Our previous study using the caloric step stimulus test also demonstrated shortened VOR_cs_ time constants in patients with spinocerebellar degeneration (16). The present study aimed to assess the clinical utility of the caloric step stimulus test for assessing patients with peripheral vestibular disorders by calculating the time constants of VOR_cs_ and VOR_cs_ adaptation and comparing these values between patients and normal controls, as well as between affected and healthy ears.

## Materials and Methods

### Subjects

This study enrolled 12 normal controls (23 ears, one subject only wanted to be tested in one ear) who were unpaid volunteers recruited in the hospital, and 74 patients (148 ears) with sudden idiopathic hearing loss (SIHL), vestibular neuritis (VN), Meniere disease (MD), vestibular Meniere disease (VMD), or chronic unilateral idiopathic vestibulopathy (CUIV), between April 2017 and June 2018 at a single tertiary center (Table 1). None of the participants had diseases of the external ear canal or the middle ear. Six otolaryngologists diagnosed each patient according to the International Classification of Vestibular Disorders of Bárány Society (17, 18), International Classification of Diseases 11th Revision (19), and diagnostic standardization guidelines established by the Japan Society for Equilibrium Research (20, 21). All patients with VN were tested within 1 month of onset. Patients presenting vestibular symptoms of MD (e.g., two or more episodes of vertigo or dizziness that lasted between 20 min and 12 h) but lacking fluctuating aural symptoms were diagnosed with VMD according to the Japanese diagnostic criteria (20). CUIV was defined by the presence of a vestibular symptom of uncertain origin that lasted more than 3 months and a spontaneous direction-fixed horizontal nystagmus. The affected ear was determined by the presence of aural symptoms or direction of nystagmus. Patients with central nervous system disorders or cardiovascular failure were excluded from this study. Patients with benign paroxysmal positional vertigo were also excluded from this study because the caloric step stimulus test involves head position changes, complicating the distinction between nystagmus caused by the test and that caused by benign paroxysmal positional vertigo.

**Table 1:** Number of subjects and ears.

	**Enrolled**		**Data analyzed**		**Data analyzed in both ears**	
**Disease**	**Subjects**	**Ears**	**Subjects**	**Ears**	**Subjects**	**Ears**
SIHL	14	28	11	16	5	10
VN	14	28	10	17	6	12
MD	13	26	11	18	6	12
VMD	9	18	9	17	8	16
CUIV	24	48	21	33	12	24
Normal controls	12	23	12	15	3	6
Total	86	171	74	116	40	80

*SIHL, Sudden Idiopathic Hearing Loss; VN, Vestibular Neuritis; MD, Meniere disease; VMD, vestibular Meniere disease; CUIV, Chronic Unilateral Idiopathic Vestibulopathy*.

This study was approved by the ethical committee of Tsuchiura Kyodo General Hospital and was conducted in accordance with the Declaration of Helsinki. Written consent was obtained from all participants after they received detailed explanations of the study procedures.

### Caloric Step Stimulus Test

**Figure 1:**
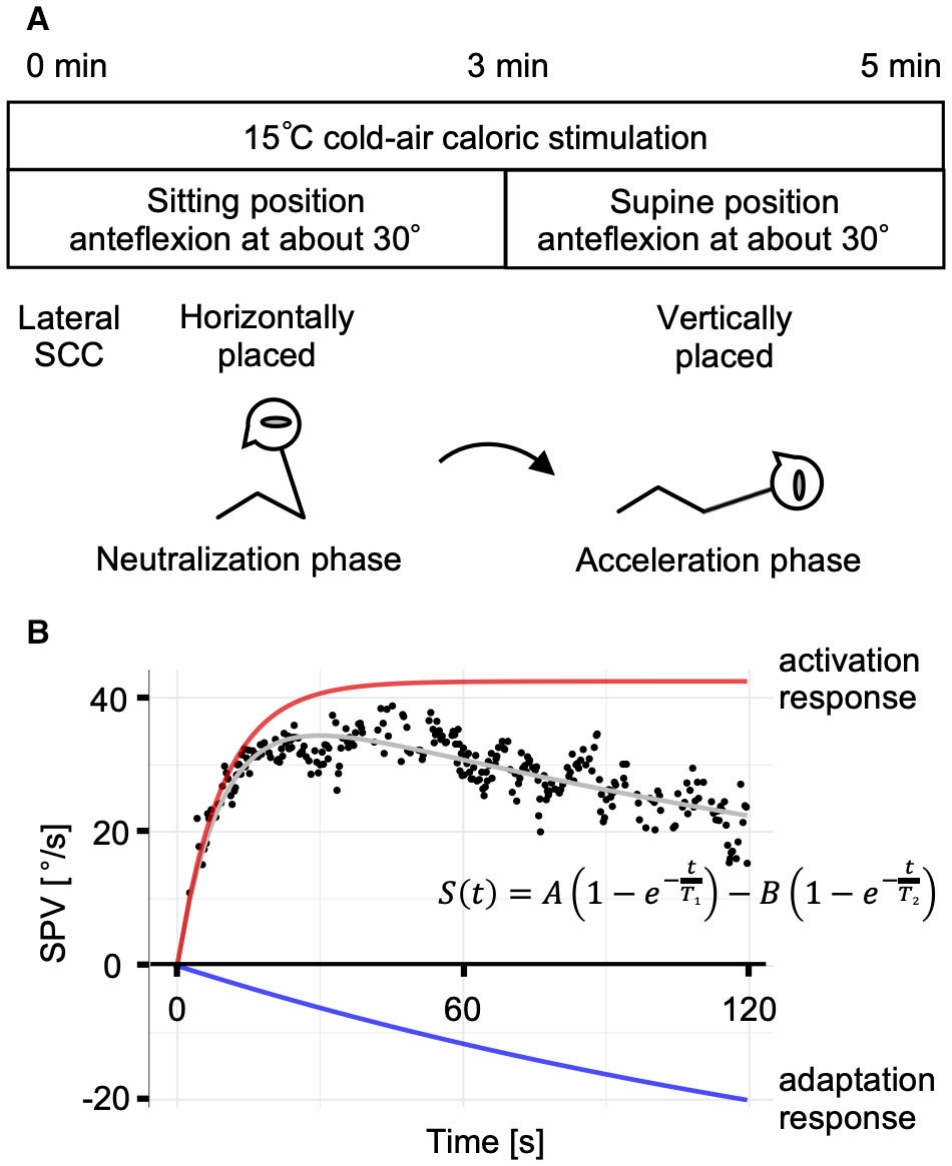
The caloric step stimulus test and the mathematical model. **(A)** A schematic representation of the caloric step stimulus test. Air caloric stimulation at 15°C is administered for a total of 5 min. The subject's lateral semicircular canal (SCC) is kept horizontal in the sitting position for 3 min (the neutralization phase), which is followed by a quick transition to the supine position to place the lateral SCC vertically. After the head position change, slow-phase eye velocity (SPV) is recorded for 2 min (the acceleration phase). **(B)** A representative response of a normal control and the mathematical model of the vestibulo-ocular reflex (VOR) during the acceleration phase. The plot of SPV vs. time is described and curve-fitted to the summation model of the activation and adaptation responses. The strength of adaptation (coefficient *B*) was considered equal to the value of activation (coefficient *A*). A: amplitude of the activation response; B: amplitude of the adaptation response; *T*_1_, the caloric step VOR (VOR_cs_) time constant; *T*_2_, the VOR_cs_ adaptation time constant.

The caloric step stimulus test was performed as previously described (16, 22). Briefly, caloric irrigation was performed with cold air (15°C, 6 L/min) using an air caloric stimulator (FAC-700, Dai-Ichi Medical Co., Ltd., Japan). The subject underwent caloric irrigation in a sitting position with 30° of head anteflexion (the neutralization phase); the head position was adjusted forward or backward to prevent the appearance of horizontal nystagmus, and 3 min after irrigation onset, the subject was rapidly (within 1 s) moved to a supine position with 30° of head anteflexion. The air caloric irrigation was continued during the next 2 min in the supine position (the acceleration phase) for a total of 5 min of stimulation (Figure 1A). We set the acceleration phase to 2 min because longer stimulation would be distressing for subjects. The maximum values of slow-phase eye velocity (SPV) of normal subjects were observed within 1 min in our previous reports. We considered that the acceleration phase for 2 min was sufficient to observe VOR responses. Stimulation began with the affected ear for patients and with either the right or left ear at random for normal controls. After testing one ear, subjects were kept in the supine position for approximately 5 min before the other ear was stimulated.

### Eye Movement Recording and Analysis

Eye movement was recorded with videonystagmography (VNG) (VN415/VO425, Interacoustics a/s, Denmark). SPV was calculated by a software that accompanied the VNG system.

The VOR response in the caloric step stimulus test was assumed as the summation of the activation and adaptation components using the following equation (14):

$$\begin{array}{l} S\left(t\right)=A\;\left[1-e^{\left(-\frac{t}{T_{1}}\right)}\right]-B\;\left[1-e^{\left(-\frac{t}{T_{2}}\right)}\right] \end{array}$$

where the coefficients *A* and *B* [°/s] represent the strengths of the activation and adaptation components; *T*_1_, the VOR_cs_ time constant; and *T*_2_, the VOR_cs_ adaptation time constant. The timeline was initiated when the subject moved to the supine position (*t* = 0). The first half of the equation (activation response) represents the rapid rise and persistence of the VOR response induced by the caloric step stimulus test. The latter half (adaptation response) represents the gradual decay of the VOR response (Figure 1B). In this study, *B* was assumed to equal *A* to reduce the number of parameters calculated by non-linear least squares. This assumption was informed by data obtained in previous studies (22).

The SPV data obtained in each ear were analyzed in the following steps:

1. Incorrect data points due to blinking, eyelashes, or roving were removed manually by examining the videos.
2. Ears were excluded according to the following criteria: interruption of the recording due to nausea, the presence of nystagmus (SPV > 10°/s) in the neutralization phase, or insufficient number of data points (<25 points/60 s).
3. Outliers of the SPV data in the acceleration phase were removed using a simple moving average method. Briefly, the average SPV was calculated in a group of seven points before and after each point. Further, an absolute value obtained by subtracting the average from the SPV of each point was calculated. If this absolute value was >1.5 standard deviations (SD) of each group, it was considered an outlier.
4. The mean and maximum values of SPV in the acceleration phase were calculated (SPV_mean_ and SPV_max_). Prior to the calculation, smoothing of the SPV data was performed by the locally weighted smoothing method.
5. The time constants were calculated by non-linear least squares. Curve fitting was performed by comparing the SPV data in the acceleration phase with the mathematical model by varying each parameter to minimize the mean square error. The range specifications of the parameters were set as 5–150°/s for *A*, 1–50 s for *T*_1_, and 50–9,999 s for *T*_2_.

Unilateral weakness (UW) was calculated using the following formula:

$$\begin{array}{l} UW\left(\%\right)=|\frac{{SPV}_{max}\left(right\right)-{SPV}_{max}\left(left\right)}{{SPV}_{max}\left(right\right)\;{+\;SPV}_{max}\left(left\right)}|\times 100 \end{array}$$

UW values of ≥20% were considered abnormal. UW was calculated only for normal controls.

### Statistics and Data Processing

The data are expressed as mean ± 1 SD. Statistical analyses were performed using Fisher's exact test, the Wilcoxon rank-sum test, and the Kruskal–Wallis test. Correlations were measured with the Kendall rank correlation coefficient. Values of *p * < 0.05 were considered to indicate statistical significance. All analyses and calculations were performed using the statistical software R.

## Results

**Figure 2:**
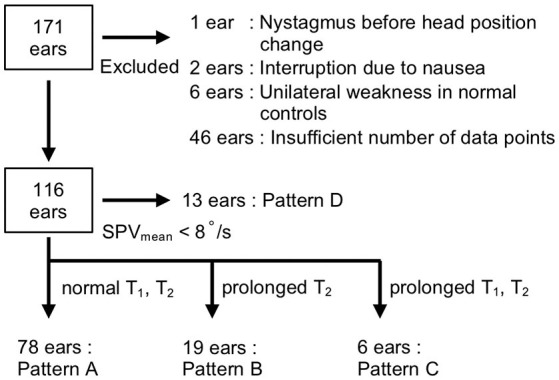
Flow diagram of the selection criteria. Ears (55 out of 171) were excluded for the following reasons: the presence of nystagmus before head position change (*n* = 1 ear), interruption due to nausea (*n* = 2 ears), unilateral weakness in normal controls (*n* = 6 ears), and insufficient number of data points (*n* = 46 ears). The responses of 116 ears were classified into four patterns according to SPV_mean_ and the time constants (*T*_1_, *T*_2_): pattern A (*T*_1_ ≤ 22 s and *T*_2_ ≤ 463 s), pattern B (*T*_1_ ≤ 22 s and *T*_2_ > 463 s), pattern C (*T*_1_ > 22 s and *T*_2_ > 463 s), and pattern D (SPV_mean_ <8°/s).

We examined a total of 171 ears; 55 (eight, normal controls; 47, patients) were excluded from further analysis (Figure 2) due to the following reasons: considerable nystagmus (SPV > 10°/s) was observed during the neutralization phase (*n* = 1 ear); tests were interrupted due to nausea (*n* = 2 ears), UW was observed in normal controls (*n* = 6 ears); and insufficient number of data points (<25 points/60 s) due to closed eyes, eyelid ptosis, or fogging of the VNG lens (*n* = 46 ears).

**Figure 3:**
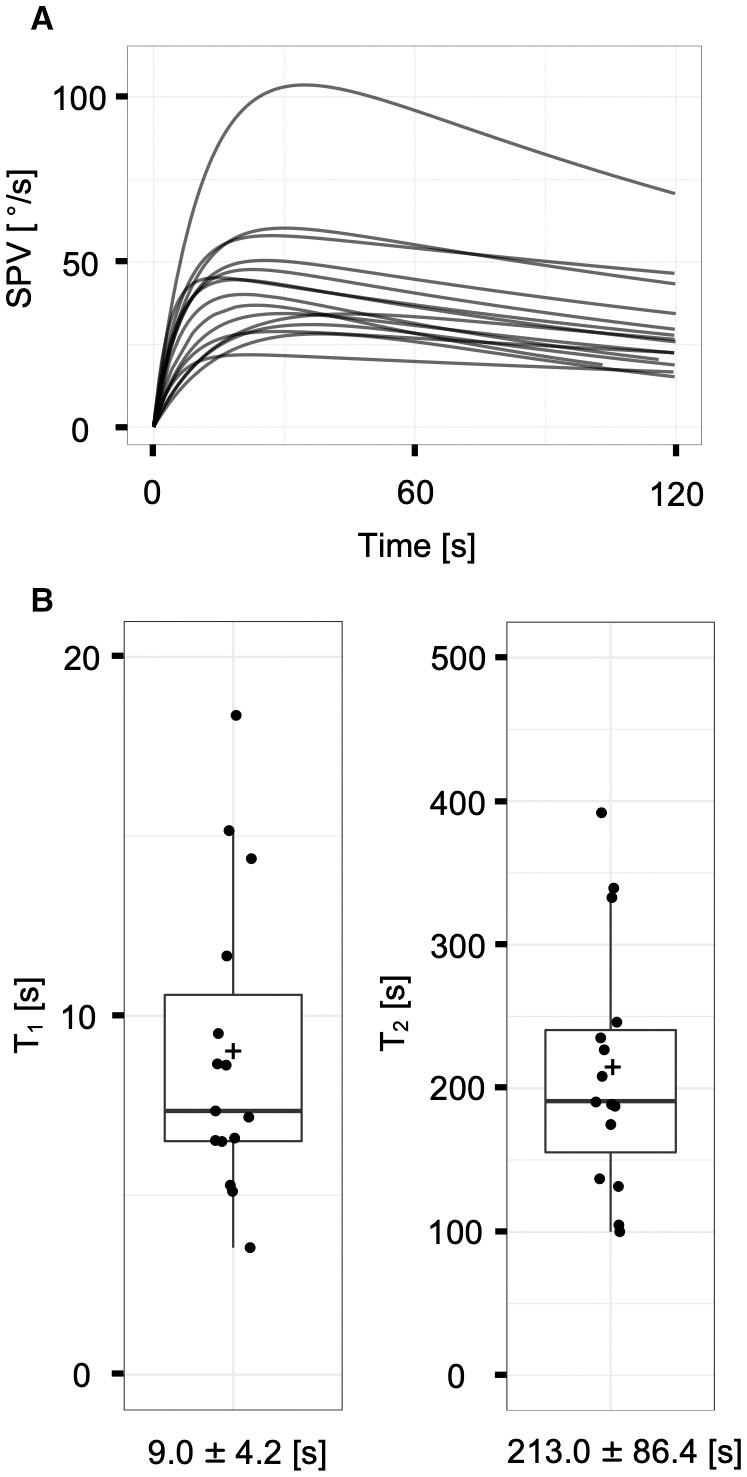
The vestibulo-ocular reflex responses of normal controls (*n* = 15 ears). **(A)** The superposition of model-fit curves of all subjects with the non-linear least square method. Each curve fit to the mathematical model appropriately. **(B)** Distributions of the time constants (*T*_1_ and *T*_2_) of normal controls. Each plus sign, solid horizontal line, and box represents the mean, median, and interquartile range, respectively. The means of *T*_1_ and *T*_2_ are 9.0 ± 4.2 and 213.0 ± 86.4 s, respectively.

The responses of the 15 ears of the normal controls were fitted to the two-time constant model (Figure 3A). SPV_max_ was 46.4 ± 20.7°/s (range: 23.0–108.2°/s). The estimated VOR_cs_ and VOR_cs_ adaptation time constants were 9.0 ± 4.2 s (range: 3.5–18.4 s) for *T*_1_ and 213.0 ± 86.4 s (range: 100.0–392.0 s) for *T*_2_ (Figure 3B). The values of *T*_1_ and *T*_2_ for normal controls in the present study were generally consistent with those reported in our previous studies (16, 22), indicating that our protocol and recording system could provide reliable data.

**Figure 4:**
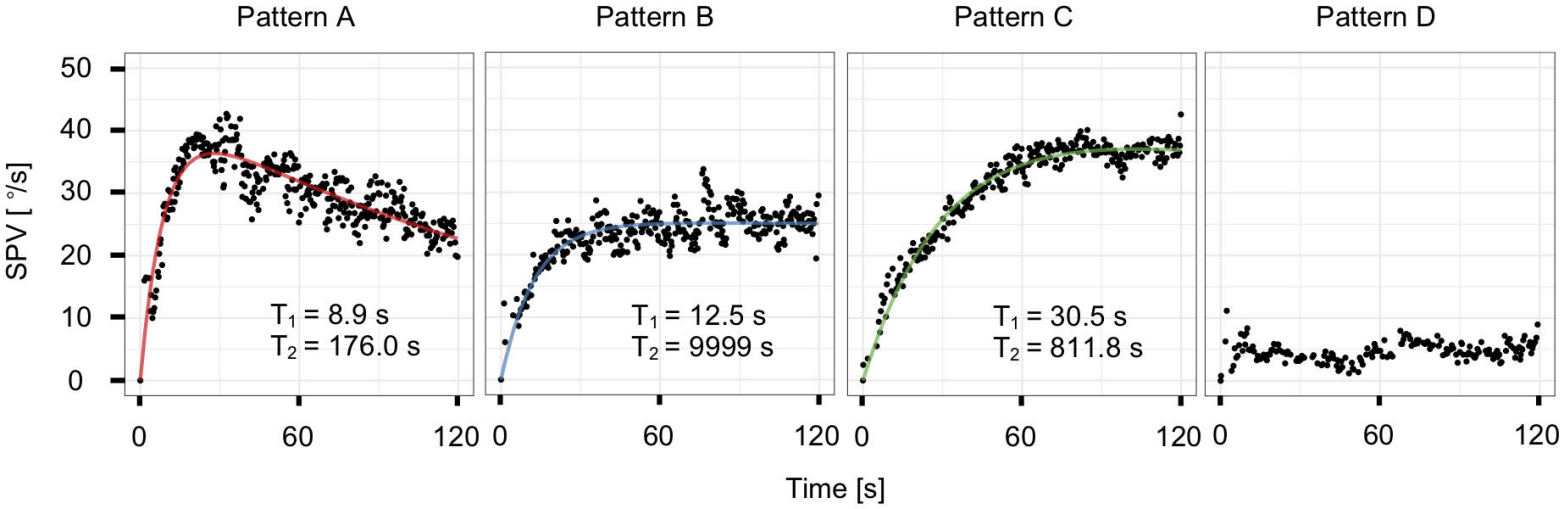
Representative plots and model-fit curves of the four patterns from a single subject: rapid rise with decay (pattern A), rapid rise without decay (pattern B), slow rise (pattern C), and low response (pattern D).

By contrast, as the responses of the patients (*n* = 101 ears) followed a variety of patterns, we categorized the responses of the 116 ears of patients and normal controls into four patterns according to the values of the SPV_mean_ and time constants: rapid rise with decay (pattern A), rapid rise without decay (pattern B), slow rise (pattern C), and low response (pattern D) (Figures 2, 4). The thresholds for classification were as follows: SPV_mean_, 8°/s; *T*_1_, 22 s; and *T*_2_, 463 s. We defined the threshold of SPV_mean_ as the minimum value at which the time constants could be calculated by curve fitting. We defined the threshold of each time constant as mean + 3 SD of normal controls. When both time constants were within normal limits (*T*_1_ ≤ 22 s and *T*_2_ ≤ 463 s), the responses were classified as pattern A (*n* = 78 ears, Supplemental Figure 1). All pattern A responses were fitted by the mathematical model. The estimated time constants were *T*_1_ = 8.0 ± 3.6 s and *T*_2_ = 205.4 ± 97.3 s; the time constants of the patients categorized into pattern A (*n* = 63 ears) did not differ significantly from those of normal controls (*n* = 15 ears; *p* = 0.40 for *T*_1_ and *p* = 0.53 for *T*_2_, Wilcoxon rank-sum test). When *T*_1_ was normal (*T*_1_ ≤ 22 s) and *T*_2_ exceeded the upper normal limit (*T*_2_ > 463 s), the responses were classified as pattern B (*n* = 19 ears, Supplemental Figure 2). In this pattern, the VOR response increased rapidly after the head position change in the same manner as pattern A, but with weak or no decay. The estimated time constants were *T*_1_ = 8.0 ± 5.3 s and *T*_2_ = 5,621.1 ± 4,427.1 s. Although the large time constants of *T*_2_ were biologically implausible, we considered the parameter as a qualitative factor that indicated the absence of adaptation when *T*_2_ exceeded the threshold. When both time constants exceeded the upper normal limits (*T*_1_ > 22 s and *T*_2_ > 463 s), the responses were classified as pattern C (*n* = 6 ears, Supplemental Figure 3). In this pattern, the VOR response increased slowly, and there was no decay. The estimated time constants were *T*_1_ = 64.2 ± 58.9 s and *T*_2_ = 8,467.8 ± 3,750.7 s. Fourth, when SPV_mean_ was <8°/s, the responses were classified as pattern D (*n* = 13 ears, Supplemental Figure 4). In this pattern, the time constants could not be obtained due to low SPV.

We subsequently sought to identify differences in the distribution of the four patterns, SPV_max_, *T*_1_, and *T*_2_, between according to disease and the affected ear (Table 2, Supplemental Figure 5). We divided the 116 ears into 11 groups and performed comparisons between the ears of patients with each disease and those of normal controls, as well as between the affected and healthy ears of the patients with each disease. There were no significant differences in the distributions of the four patterns and SPV_max_ between any two groups, except between the affected and healthy ears of VN (Table 2, *p* < 0.05, Fisher's exact test; *p* < 0.01, Wilcoxon rank-sum test). In terms of time constants, there was no significant difference in *T*_1_ or *T*_2_ between the affected and healthy ears in patients with each disease (Table 2, Wilcoxon rank-sum test). No significant differences were found between the ears of the patients with each disease and those of normal controls (Table 2, *p* = 0.47 for *T*_1_ and *p* = 0.094 for *T*_2_, Kruskal–Wallis test). However, pattern C was observed only in six affected ears of patients with peripheral vestibular disorders.

**Table 2:** Number of ears that followed each pattern, SPV_max_ and time constants (*T*_1_, *T*_2_) in patients and normal controls.

**Disease**		**Pattern (*****N*** **of ears)**					**Data**					
		**A**	**B**	**C**	**D**	***p*-value**	**SPV_**max**_ (^°^/s)**	***p*-value**	***T*_**1**_ (s)**	***p*-value**	***T*_**2**_ (s)**	***p*-value**
SIHL	Affected	4	0	1	3		19.7, 11.9		7.5, 8.9		2,183.5 ± 4,370.3	
	Healthy	6	1	0	1	0.41	28.2, 16.7	0.23	5.8, 2.1	0.34	550.8 ± 976.9	0.53
VN	Affected	2	1	1	4		10.0, 7.8		42.0, 72.1		3,067.2 ± 4,672.4	
	Healthy	7	2	0	0	0.022 *1	32.9, 12.8	0.00033 *2	6.9, 3.1	0.71	1,424.5 ± 3,232.5	0.35
MD	Affected	4	3	1	1		29.5, 11.1		10.5, 9.2		2,774.8 ± 4,465.3	
	Healthy	8	1	0	0	0.16	39.0, 17.2	0.22	6.6, 3.2	0.48	229.6 ± 126.1	0.092
VMD	Affected	3	3	1	1		31.6, 17.0		26.2, 45.9		3,294.9 ± 4,613.4	
	Healthy	7	2	0	0	0.29	45.6, 20.2	0.17	10.3, 2.7	0.83	1,351.6 ± 3,250.7	0.24
CUIV	Affected	8	3	2	3		24.1, 11.0		11.2, 7.5		3,251.8 ± 4,683.5	
	Healthy	14	3	0	0	0.066	31.8, 12.5	0.10	9.0, 3.8	0.68	1,694.3 ± 3,427.1	0.19
Normal controls	Healthy	15	0	0	0		46.4, 20.7		9.0, 4.2		213.0 ± 86.4	

SIHL, Sudden Idiopathic Hearing Loss; VN, Vestibular Neuritis; MD, Meniere disease; VMD, vestibular Meniere disease; CUIV, Chronic Unilateral Idiopathic Vestibulopathy; SPV, slow-phase eye velocity; ^*^1, p <0.05 in Fisher's exact test; ^*^2, p <0.05 in Wilcoxon rank sum test.

**Figure 5:**
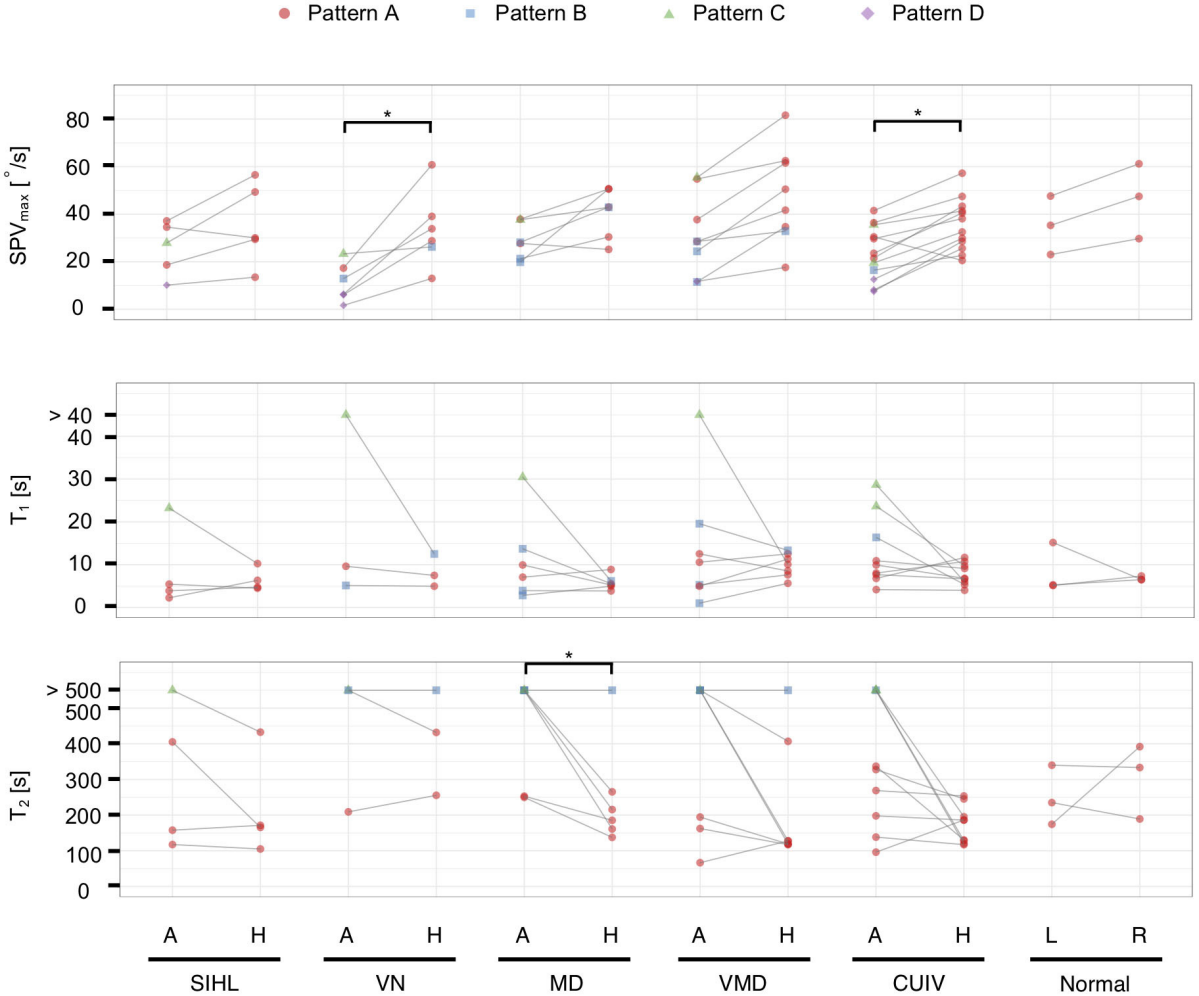
The vestibulo-ocular reflex response patterns and parameters in 40 subjects with analyzable data in both ears (*n* = 80 ears). Solid lines connect both ears in the same person. For *T*_1_ and *T*_2_, boxplots were created from datapoints without those that exceeded the upper limits (*T*_1_ > 22 s, *T*_2_ > 463 s). Significant differences in SPV_max_ were found between the affected and healthy ears in VN and CUIV. Prolongation of *T*_1_ was observed in only six affected ears. Prolongation of *T*_2_ was observed significantly more frequently in the affected ears than the healthy ears of patients with MD. A, the affected ear; H, the healthy ear; R, right ear; L, left ear; *p < 0.05 in Wilcoxon rank-sum test.

We also performed comparisons between the affected ears (the right ear in normal controls) and healthy ears (the left ear in normal controls) in the same person (Figure 5). The data were analyzable in both ears in 40 subjects (*n* = 80 ears). Regarding the distribution of the four patterns, we found significant differences between the affected and healthy ears of patients with VN and CUIV (Figure 5, *p* < 0.05 and 0.05, Fisher's exact test). There were significant differences in SPV_max_ between the two ears of patients with VN and CUIV (Figure 5, *p* < 0.05 and 0.05, Wilcoxon rank-sum test). Prolongation of *T*_2_ was significantly more frequently among the affected ears of patients with MD than the affected ears of patients with other conditions (Figure 5, *p* < 0.05, Wilcoxon rank-sum test). Prolongation of *T*_2_ was observed in three healthy ears of patients with VN, MD, VMD, all of which were accompanied by prolongation of *T*_2_ of the corresponding affected ears.

Furthermore, we compared *T*_1_ and *T*_2_ between the affected and healthy ears of patients whose time constants could be calculated. Prolongation of *T*_1_ and *T*_2_ were significantly more frequent on the affected ears than on the healthy ones in 88 ears that followed patterns A, B, or C (Supplemental Figure 5, Table 3; *p* < 0.05 and 0.05, Fisher's exact test), as well as in 58 ears that followed patterns A, B, or C (Figure 5, Table 4; *p* < 0.05 and 0.05, Fisher's exact test).

**Table 3:** Biased distribution of prolonged time constants of the affected ears of the 59 patients that were categorized as following patterns A, B, or C.

	**Affected ear**	**Healthy ear**	***p*-value**
Normal *T*_1_	31	51	
Prolonged *T*_1_	6	0	0.0043^*^
Normal *T*_2_	21	42	
Prolonged *T*_2_	16	9	0.016^*^

* *p <0.05 in Fisher's exact test*.

**Table 4:** Biased distribution of prolonged time constants of the affected ears of 29 patients whose data were available for both ears and were categorized as following patterns A, B, or C.

	**Affected ear**	**Healthy ear**	***p*-value**
Normal *T*_1_	23	29	
Prolonged *T*_1_	6	0	0.023^*^
Normal *T*_2_	15	26	
Prolonged *T*_2_	14	3	0.0032^*^

* *p <0.05 in Fisher's exact test*.

Examining the correlation between age and time constants in normal controls and the healthy ears of the patients, we found no relationship (*T*_1_: tau = −0.052, *p* = 0.54; *T*_2_: tau = −0.053, *p* = 0.53; Kendall rank correlation coefficient).

## Discussion

In our previous studies, we reported the features of VOR dynamics in normal controls (22) and patients with spinocerebellar degeneration (16) using the caloric step stimulus test. The present study is the first to evaluate VOR_cs_ and VOR_cs_ adaptation of affected and healthy ears in patients with peripheral vestibular disorders using the caloric step stimulus test. We demonstrated that the VOR_cs_ responses could be divided into four distinct patterns in the 15 ears of normal controls and the 101 ears of patients with peripheral vestibular disorders. In pattern A, the response was fitted to the mathematical model based on the results of rotational testing (10), and the VOR_cs_ and VOR_cs_ adaptation time constants (*T*_1_ and *T*_2_) of patients with peripheral vestibular disorders were similar to those of normal controls. In pattern B, a rapidly rising response without decay was observed, in which *T*_2_ was larger than the upper limit established by referencing the findings in normal controls. In pattern C, a slowly rising response in which both *T*_1_ and *T*_2_ were larger than the upper limits was observed. In pattern D, the VOR response was too low for the time constants to be calculated.

Pattern D is likely to correspond to a severe unilateral peripheral vestibular hypofunction (canal paresis) which is detectable by the conventional caloric test. On the other hand, Patterns B and C are responses that cannot be observed by the conventional caloric test and can only be detected by the caloric step stimulus test. Prolongation of *T*_1_ in pattern C was observed only in the affected ears of patients with peripheral vestibular diseases. Prolongation of *T*_2_ in patterns B and C was more frequently in the affected ears than in the healthy ones. Our results suggest that the caloric step stimulus test is capable of detecting unusual VOR responses which might reflect dysfunction of VOR, adaptation, or other pathological changes in peripheral vestibular disorders.

The presently observed averaged VOR_cs_ time constant of normal controls (9.0 ± 4.2 s) is similar to those reported in our previous studies [11.7 ± 5.1 s (16) and 10.1 ± 3.0 s (22)], but smaller than that reported by Formby and Robinson [(14) 14.1 ± 7.7 s]. This discrepancy might be attributable to the use of different caloric stimulation methods. In our studies, we used 15 or 20°C cold air irrigation for stimulation, whereas Formby and Robinson used water irrigation between 28 and 43°C in temperature. They measured the time constants for five normal controls by using multiple stimulations with water of different temperatures. The reported values featured considerable variation even in the same person. It is believed that air caloric irrigation supplies a more stable temperature stimulus than does water caloric irrigation, as water spillage can be avoided during postural changes (22).

The VOR_cs_ time constant of normal controls in this study was smaller than the VOR time constant measured by the acceleration step stimulus in rotational testing [16.0 s (9), 21.0 s (10), and 20.0 s (11)]. One plausible explanation for the decreased VOR_cs_ time constant is the participant's awareness that their body is not rotating in reality, causing a partial loss of the velocity-storage mechanism (14). Another explanation is that the static head position influences the VOR time constant. Tilting of the head forward or laterally during rotational testing decreases the VOR time constant likely because of the disengaging or dumping activity in the velocity-storage mechanism through inputs from the otolith organs (23). The supine position in the caloric step stimulus test might cause the tilt suppression effect, leading to the insufficient prolongation of the time constant in central processing.

Previous studies in rotational testing have reported that the VOR time constant is shorter upon stimulation toward the affected ear of patient's peripheral vestibular disorders, such as vestibular neuritis, Meniere disease, and unilateral vestibulopathy, after surgical resection of an acoustic neuroma (24–27). This phenomenon is thought to be attributable to the peripheral and central vestibular system. Pathological changes in the SCC may decrease the cupula time constant, which decreases the behavioral VOR time constant. The cupula time constant is inversely proportional to the stiffness of the cupula (28). The viscosity of the endolymph and afferent nerve responses could also affect the cupula time constant (8, 29). In the central vestibular system, a loss of the velocity storage mechanism in the affected ear's canal input decreases the VOR time constant, which may compensate for unilateral vestibular lesions (23, 24, 26, 27). Unlike with rotational testing, our study did not find smaller VOR_cs_ time constants in patients with peripheral vestibular disorders relative to normal controls. A possible explanation for this is that since the VOR_cs_ time constant is decreased even in normal controls, as described above, we could not detect a significant difference between peripheral vestibular disorders and normal controls. Another explanation is that rotational testing evaluates the sum of bilateral labyrinthine VOR whereas the caloric step stimulus test shows unilateral labyrinthine VOR, which may cause the difference of the feature in VOR time constant of patients between the two tests.

By contrast, significant prolongations of VOR_cs_ time constants were observed in only six of the affected ears of patients with peripheral vestibular disorders (Table 2, Figure 5, and Supplemental Figure 5). Little is known about the association between vestibular disorders and prolonged VOR time constants. Some studies have demonstrated a relationship between the level of motion sickness and the extension of the VOR time constant (30–33). In addition, lesions affecting the cerebellar nodulus and uvula maximize the velocity-storage mechanism and increase the VOR time constant in monkeys and humans. We speculate that prolongations of VOR_cs_ time constants can be derived from not only central process but also pathological alteration in the peripheral vestibular system. It was reported that the cupula time constant was proportional to the viscosity (28). If the viscosity becomes higher, the cupula time constant will be prolonged, which results in prolongation of VOR_cs_ time constant.

The VOR responses in the ears of several patients did not exhibit the decay trend, which was expressed as prolongation of *T*_2_ (patterns B and C, *T*_2_ > 463 s). The prolongation of *T*_2_ was observed significantly more in the affected ears of patients, especially those with MD. The prolongation of *T*_2_ may reflect a decreased or delayed short-term adaptation. The mechanism of VOR adaptation impairment remains uncertain. A recent study of rotational testing in mice suggested that VOR adaptation was impaired with age and the major pathophysiology was the decreased number of Purkinje cells in the cerebellum (34). However, as reported previously (16), the present study found no correlation between age and the VOR_cs_ adaptation time constant. Central process in vestibular compensation may affect VOR adaptation response. After acute unilateral vestibular loss, central vestibular compensation works for the reacquisition of body balance. The compensation of static vestibular deficits coincides with a rebalancing of firing activity between vestibular nuclei neurons of the affected side and those of the healthy side. Conversely, the compensation of dynamic vestibular deficits depends on global reorganization of the central nervous system (35, 36). We speculate that the activities of the vestibular nuclei neurons are dynamically modulated during the compensation process, leading to sustained VOR response and prolongation of *T*_2_. Alternatively, VOR adaptation impairment may occur at peripheral levels. Adaptation response to constant cupula displacement is observed in the measurement of action potential discharge rates at the peripheral afferent neurons (5, 6). Structural changes in the duct, ampulla, or cupula, or modulation of signal processing from hair cells to afferent neurons might cause the decreased or delayed adaptation response. It was pointed out that the enlarged membranous duct results in decreased caloric response in MD patients (37). The increased duct diameter may influence hydrodynamics of the endolymph in caloric stimulation which can explain prolonged *T*_2_ in the affected ears of MD patients in this study, although the underlying mechanism is unclear. Further studies are needed to elucidate the underlying pathogenesis of the usual VOR responses observed in this study.

### Limitations

Our study is subject to several limitations. First, it does not experimentally demonstrate that the caloric step stimulus test is truly equivalent to constant head acceleration. Although we assume that the stimulus effects the constant displacement of the cupula, the actual behavior may be more complex than the theoretical one. Heat transfer in the caloric test is potentially affected by the heat conductivity of the skin, blood flow, and temporal bone anatomy, such as the volume or surface area of the external canal, mastoid airspace, mastoid bone, and prominence of the lateral SCC (38). In addition, Peterka et al. (39) suggested that the dynamic response of the caloric step stimulus test is involved in not only horizontal VOR represented by the SCC but also linear VOR represented by the otolith organs. The development of a comprehensive model that accounts for heat transmission from the irrigation medium to the SCC, endolymph fluid dynamics, the direction of gravity, and canal-otolith central interaction is warranted. Second, our simplified model that assumes that coefficient *A* equals *B* does not explain incomplete adaptation observed in the long-term caloric stimulation (40) or magnetic field stimulation (13). The new adaptation model involving multiple adaptation process has been proposed to address incomplete adaptation for the long-term labyrinth stimulation (13). While the 2-min recording obtained in our study presumably measures short-term adaptation, it is insufficient for the precise measurement of the adaptation process. Third, we lacked data about their vestibular functions such as the conventional caloric test, rotational testing, head impulse test, and vestibular evoked myogenic potential testing. Fourth, non-linear curve fitting may result in significant errors in estimating the parameters. For example, the actual prolongation of *T*_1_ may affect curve-fitting calculations and lead to pseudo-prolongation of *T*_2_ or vice versa. Fifth, there were 55 ears that dropped out of the analysis. Forty-six ears were dropped out due to insufficient number of data points, which was caused by the failure to identify subjects' pupil by VNG (Figure 2). If we used the device with better pupil detection or electronystagmography, the lack of pupil detection due to closed eyes or eyelid ptosis could be prevented.

## Conclusions

The VOR_cs_ and VOR_cs_ adaptation time constants of peripheral vestibular disorders were measured using the caloric step stimulus test. The prolongation of the VOR_cs_ or VOR_cs_ adaptation time constants were observed in the affected ears of patients, suggesting that the caloric step stimulus test, unlike the conventional caloric test, can provide insight into the presence of pathological changes.

## Data Availability Statement

The raw data supporting the conclusions of this article will be made available by the authors, without undue reservation.

## Ethics Statement

The studies involving human participants were reviewed and approved by the ethical committee of Tsuchiura Kyodo General Hospital. The patients/participants provided their written informed consent to participate in this study.

## Author Contributions

MH contributed research design, data collection, data processing, making figures, and writing the manuscript. KH contributed research design, data processing, and writing the manuscript. TT supervised the study and contributed to the review and editing of the manuscript. All authors read and approved the final manuscript.

## Conflict of Interest

The authors declare that the research was conducted in the absence of any commercial or financial relationships that could be construed as a potential conflict of interest.
