# Supplementary material for: Unusual Vestibulo-Ocular Reflex Responses in Patients With Peripheral Vestibular Disorders Detected by the Caloric Step Stimulus Test
Source: Front Neurol. 2020 Nov 30;11:597562. doi: 10.3389/fneur.2020.597562 (PMC7734291; doi:10.3389/fneur.2020.597562)
Supplement: Supplementary file 1 [file Image_1.PDF]

Supplemental figure

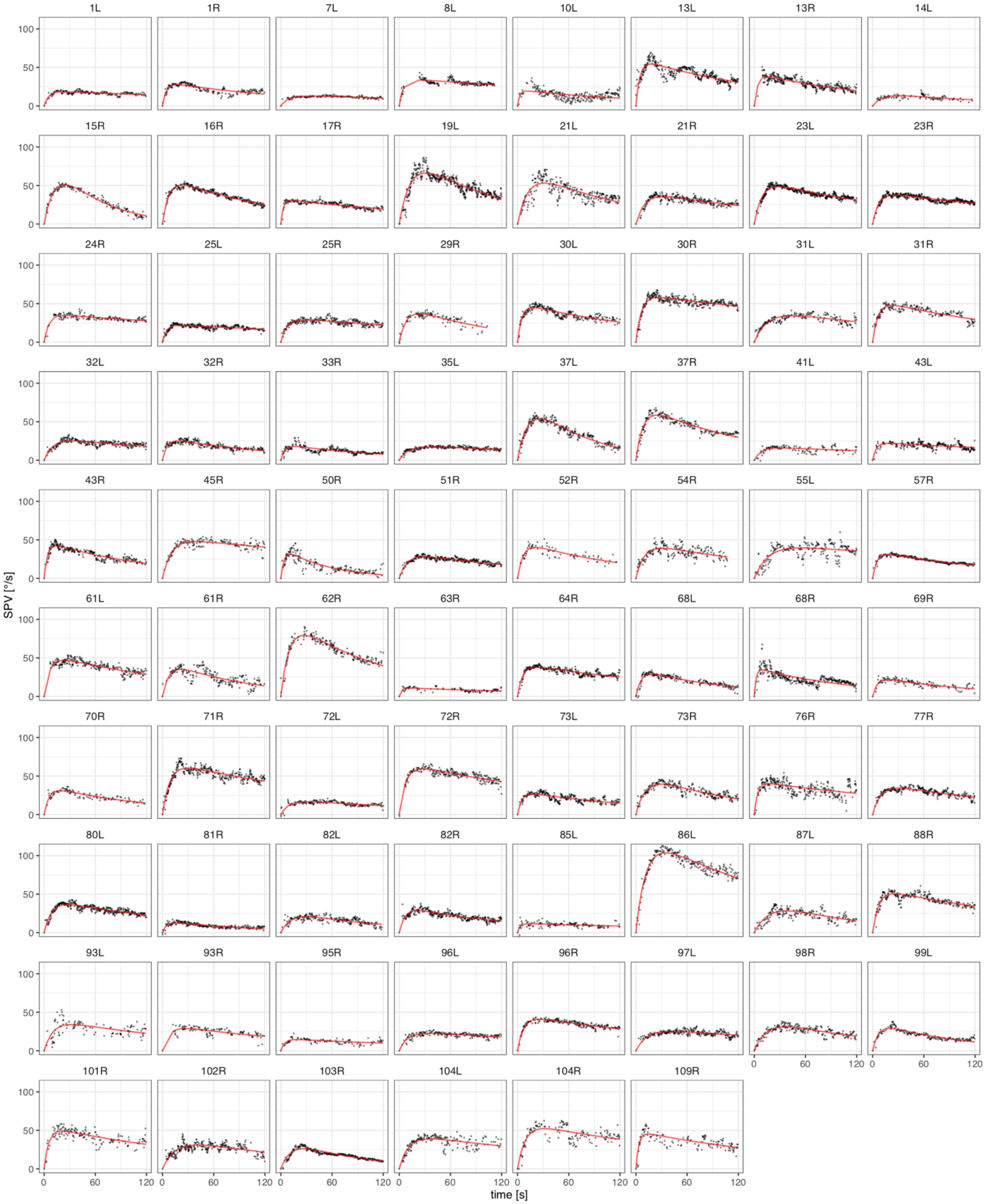

Supplemental figure 1. The plots of slow-phase eye velocity (SPV) vs. time and model-fit curves of all 78 subjects in pattern A. The subject number and tested ear (R: right ear, L: left ear) are above each graph. The trajectory of SPV formed a rapid rise with decay. The means of T_1 and T_2 were 8.0 ± 3.6 s and 205.4 ± 97.3 s.

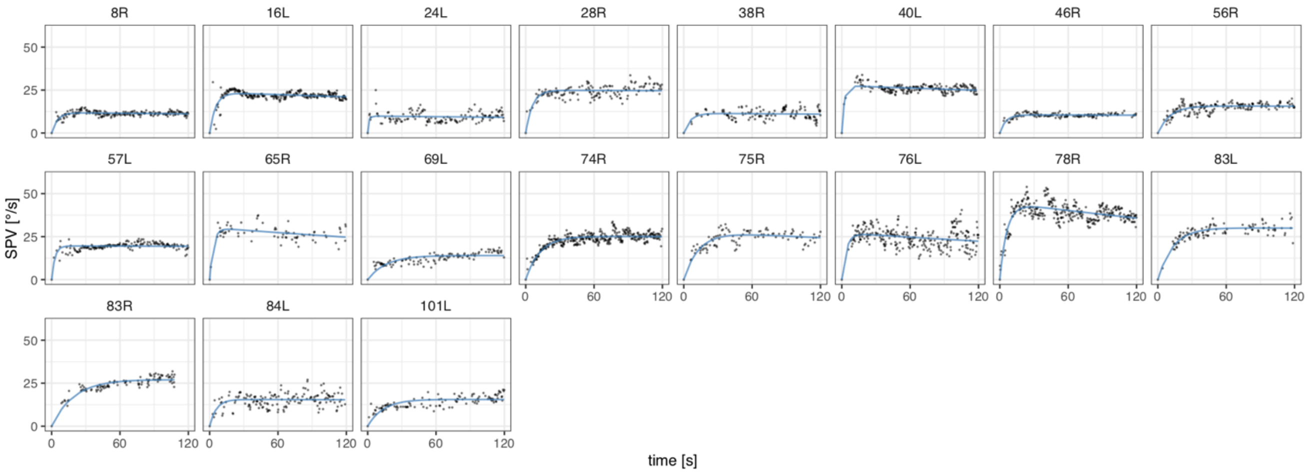

Supplemental figure 2. The plots of SPV vs. time and model-fit curves of all 19 subjects in pattern B. The subject number and tested ear (R: right ear, L: left ear) are above each graph. The trajectory of SPV formed a rapid rise without decay. The means of T_1 and T_2 were 8.0 ± 5.3 s and 5621.1 ± 4427.1 s.

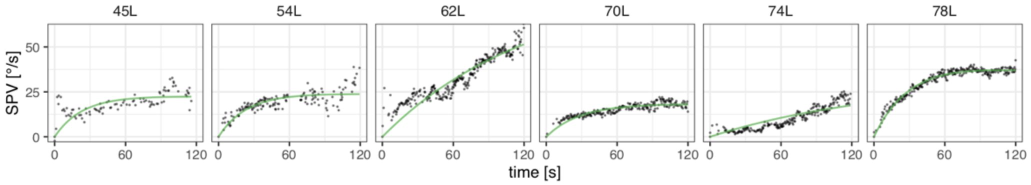

Supplemental figure 3. The plots of SPV vs. time and model-fit curves of all 6 subjects in pattern C. The subject number and tested ear (R: right ear, L: left ear) are above each graph. The trajectory of SPV formed a slow rise. The means of T_1 and T_2 were 64.2 ± 58.9 s and 8467.8 ± 3750.7 s.

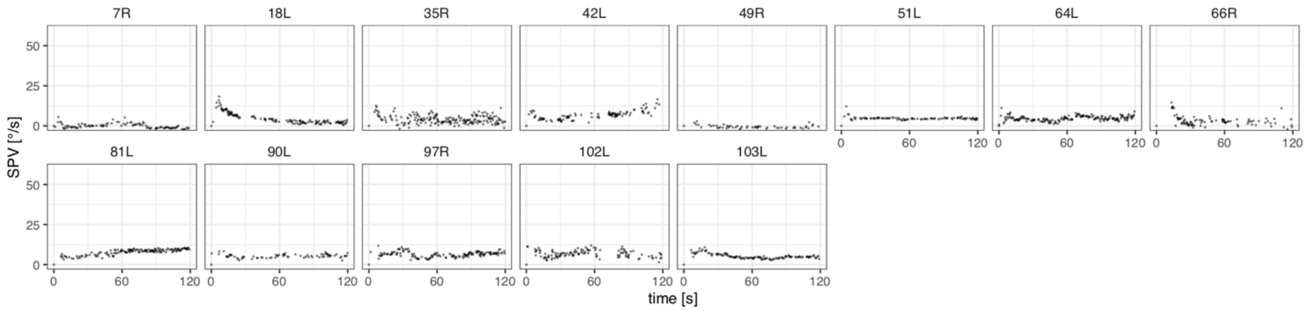

Supplemental figure 4. The plots of SPV vs. time of all 13 subjects in pattern D. The subject number and tested ear (R: right ear, L: left ear) are above each graph. Each subject exhibited low VOR response. The time constants could not be obtained due to low SPV.

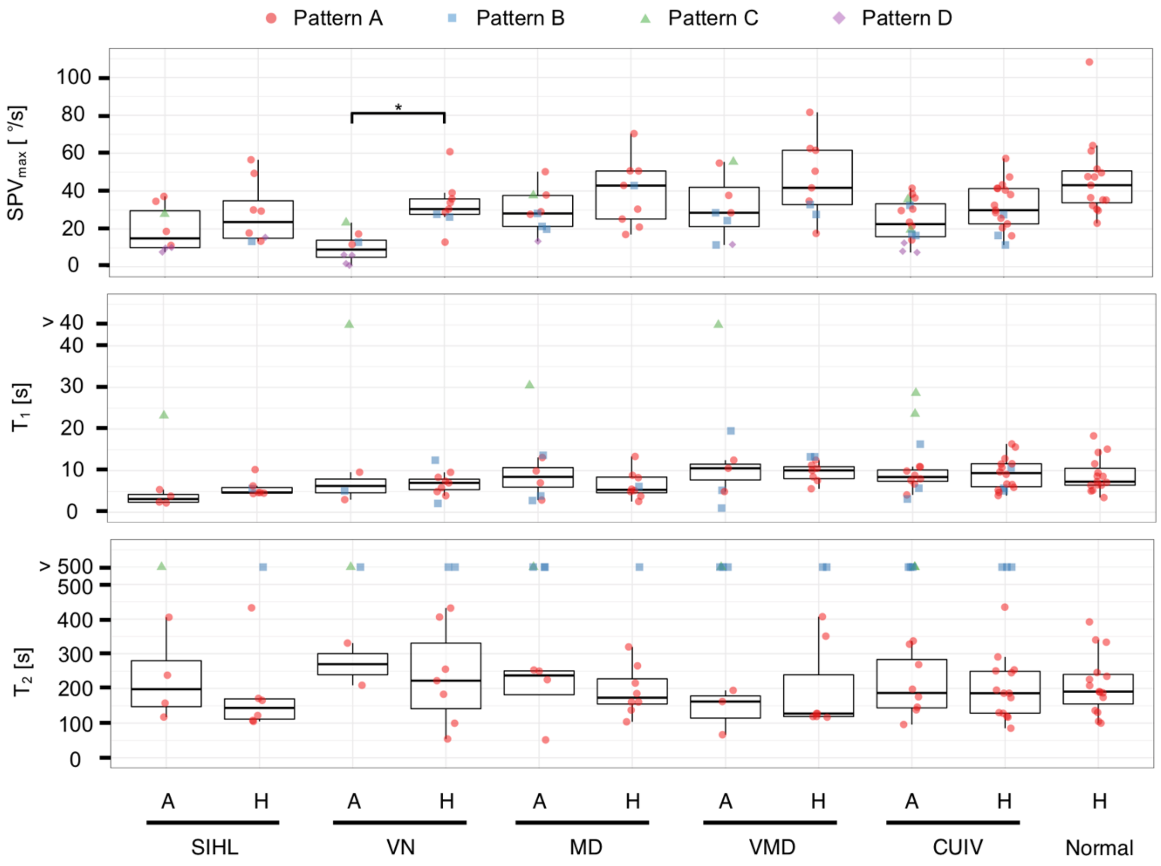

Supplemental figure 5. The breakdown of jitter plots of the estimated parameters of 116 ears' vestibulo-ocular reflex responses (top: SPV_{max}, middle: T_1, bottom: T_2). In SIHL, VN, MD, VMD, and CUIV, the results are shown separately for the affected and healthy ears. In normal controls, both ears are combined into the same group. For T_1 and T_2, boxplots were created from datapoints without those over the upper limits ($T_1 > 22$ s, $T_2 > 463$ s). There was a significant difference in the distribution of SPV_{max} between the affected and healthy ear in VN. Notably, prolongation of T_1 (pattern C) was observed only in six affected ears. A: the affected ear; H: the healthy ear; $^*p < 0.05$ in Wilcoxon rank-sum test.
